# Bullying victimization and stress sensitivity in help-seeking youth: findings from an experience sampling study

**DOI:** 10.1007/s00787-020-01540-5

**Published:** 2020-05-13

**Authors:** Christian Rauschenberg, Jim van Os, Matthieu Goedhart, Jan N. M. Schieveld, Ulrich Reininghaus

**Affiliations:** 1 Department of Psychiatry and Neuropsychology, School for Mental Health and Neuroscience, Maastricht University, Maastricht, The Netherlands; 2 Department of Public Mental Health, Central Institute of Mental Health, Medical Faculty Mannheim, University of Heidelberg, Mannheim, Germany; 3 Department of Psychiatry, Brain Centre Rudolf Magnus, University Medical Centre Utrecht, Utrecht, The Netherlands; 4 Psychosis Studies Department, Institute of Psychiatry, Psychology and Neuroscience, King’s College London, London, UK; 5 Tilburg School of Humanities, Tilburg University, Tilburg, The Netherlands; 6 Mutsaers Foundation and Educational Institute Wijnberg, Venlo, The Netherlands; 7 Department of Psychiatry and Psychology, Division of Child and Adolescent Psychiatry, Maastricht University Medical Center (MUMC+), Maastricht, The Netherlands; 8 Health Service and Population Research Department, Centre for Epidemiology and Public Health, Institute of Psychiatry, Psychology and Neuroscience, King’s College London, London, UK

**Keywords:** Bullying, Stress, Adolescent, Mental health, Psychopathology, Ecological momentary assessment

## Abstract

Bullying victimization confers the risk for developing various mental disorders, but studies investigating candidate mechanisms remain scarce, especially in the realm of youth mental health. Elevated stress sensitivity may constitute a mechanism linking bullying victimization and mental health problems. In the current study, we aimed to investigate whether exposure to bullying victimization amplifies stress sensitivity in youth’s daily life. The Experience Sampling Method (ESM) was used to measure stress sensitivity [i.e. the association of momentary stress with (i) negative affect and (ii) psychotic experiences] in 42 help-seeking youths (service users), 17 siblings, and 40 comparison subjects (mean age 15 years). Before ESM assessments, bullying victimization at school as well as various psychopathological domains (i.e. depression, anxiety, psychosis) were assessed. Service users exposed to high levels of overall (primary hypotheses) as well as specific types (secondary hypotheses; physical and indirect, but not verbal) of bullying victimization experienced more intense negative affect and psychotic experiences in response to stress compared to those with low exposure levels (all *p* < 0.05), whereas, in contrast, controls showed either less intense negative affect or no marked differences in stress sensitivity by exposure levels. In siblings, a less consistent pattern of findings was observed. Findings suggest that stress sensitivity may constitute a potential risk and resilience mechanism linking bullying victimization and youth mental health. Interventions that directly target individuals’ reactivity to stress by providing treatment components in real-life using mHealth tools may be a promising novel therapeutic approach.

**Electronic supplementary material:**

The online version of this article (10.1007/s00787-020-01540-5) contains supplementary material, which is available to authorized users.

## Introduction

Bullying victimization is defined as an intentional misuse of power in which an individual or a group of individuals engage in hostile behaviour against peers who have difficulties to defend themselves [1]. The experience of being bullied has long been seen to reflect a normal pattern of interaction between peers that is transitory and important for individuals’ social development [2]. As a consequence, exposure to bullying has not been considered to represent a particularly stressful experience and, therefore, not to be an important risk factor involved in the development of mental health problems [2, 3].

However, evidence has accumulated that exposure to bullying victimization is associated with a range of mental disorders (e.g. depression, anxiety, psychosis), general psychopathology, self-harm, and suicidality, amongst others [4–13], and has been found to predict the use of mental health services [14]. These findings suggest that exposure to bullying victimization may be an important non-specific risk factor for mental health problems, which is consistent with the detrimental, but also non-specific effects reported for other adverse childhood experiences (e.g. childhood maltreatment) [15]. Recent estimates from the World Health Organization [16] are alarming: around two in ten children and adolescents are being exposed to bullying victimization at school, although prevalence estimates differ considerably across countries (e.g. in Europe: 4% in Italy, 30% in Lithuania). These findings have contributed to formal recognition of bullying as a risk factor for mental health problems in the Global Burden of Disease Study 2017 [17].

In recent years, there has been an increasing focus on dimensional and transdiagnostic approaches to psychopathology [18–20], resulting in classification frameworks (e.g. HiTOP) [21] that are based on patterns of symptom co-occurrence, cutting across traditional diagnostic boundaries. In support of these efforts and also based on frequent co-occurrence of more common psychopathological domains (e.g. anxiety, depression) with psychotic experiences, an extended and transdiagnostic psychosis spectrum phenotype has been proposed that is temporally and phenomenologically continuous across psychotic and non-psychotic disorders and shares socio-environmental risk factors, including bullying victimization [22].

Overall, high prevalence of bullying victimization in youth, and associations with immediate as well as prolonged mental health problems, which are often characterized by a number of co-occurring psychopathological domains (e.g. anxiety, depression, psychosis) [22, 23] that appear already early in life [24] underline the importance to develop early and transdiagnostic intervention strategies [25]. For this, an important step is to investigate candidate mechanisms that are relevant to linking exposure to bullying victimization and mental health problems. Critically, however, the developmental processes and putative mechanisms involved remain largely under-researched, especially in the realm of youth mental health.

In contemporary models, exposure to socio-environmental risk (e.g., bullying victimization, childhood maltreatment, life events) is thought to impact on mental health through a progressive increase in individuals’ stress response to subsequent adversity [26, 27]. This has often been referred to as a process of sensitization [28] which is thought to be mediated by a number of biological and psychological factors [29–34]. Although evidence remains limited, there is an ongoing debate [15] regarding the extent to which specific forms of adversity may be more strongly associated with specific forms of mental health outcomes (e.g., whether more intrusive types of adversity are specifically associated with psychosis). The Experience Sampling Methodology (ESM), a structured self-report diary technique [35], is particularly well suited to test these propositions at a behavioural level by investigating whether exposure to overall as well as specific types of bullying victimization is associated with an increased sensitivity to minor stressors in daily life.

In most studies using ESM, individuals’ stress sensitivity has been conceptualized as the association of minor stressors with (i) negative affect and (ii) psychotic experiences in daily life and, thus, has also been referred to individuals’ affective and psychotic reactivity, respectively. These studies have consistently found an increased stress sensitivity in adults who were exposed to childhood trauma and adult life events, including individuals with depression, an at-risk mental state for psychosis, and psychosis spectrum disorders [36–39]. Thus, findings suggest that stress sensitivity in the flow of daily life may play an important non-specific and transdiagnostic role linking childhood adversity and mental disorders in help-seeking individuals. In a recent experience sampling study, derived from the same sample as the current study, exposure to childhood trauma was, similarly, associated with elevated stress sensitivity in help-seeking youth [40]. Also, there is some evidence of specificity as some studies have reported that more intrusive forms of childhood trauma (e.g. sexual and physical abuse as well as physical neglect) were most consistently associated with an elevated stress sensitivity in help-seeking individuals [38, 40]. Consequently, more intrusive forms of bullying (i.e., physical bullying) may be particularly associated with an increased stress sensitivity in help-seeking youth.

To date, however, only one study has reported elevated stress sensitivity in a non-clinical sample of young adults exposed to bullying [41] and, to the best of our knowledge, no study has investigated the impact of bullying victimization on individuals’ affective and psychotic reactivity to stress in a sample of help-seeking youth and whether effects of bullying exposure on stress sensitivity differ across individuals at differing liability to mental health conditions. To address current knowledge gaps, a sample of adolescents and young adults receiving help from a secondary mental health service (service users), their biological siblings, and controls were recruited in the current study. We included siblings of service users as they have an increased risk for developing a mental disorder and, hence, reflect an intermediate risk group (compared with service users and controls) and also share genetic and socio-environmental risk factors with service users [42–44].

### Aims and hypotheses

The aim of the current study was to determine whether bullying victimization modifies sensitivity towards stress in a sample of help-seeking youth (service users), their biological siblings, and comparison subjects (controls). More specifically, the study aimed to investigate the following primary hypotheses: First, within groups (service users, siblings, and controls), stress sensitivity (i.e., the association between momentary stress and (i) negative affect and (ii) psychotic experiences) is modified by bullying victimization, with greater associations in individuals exposed to high *vs.* those exposed to low exposure levels of bullying victimization (H1); second, the effect of bullying victimization on stress sensitivity differs across groups at differing liability to mental health problems, with a greater impact in service users vs*.* controls, service users vs*.* siblings, and siblings vs. controls (H2). In addition, to investigate whether some bullying types are specifically modifying stress sensitivity, the following secondary hypotheses were tested: first, within groups, exposure to specific bullying types (i.e. physical, verbal, indirect bullying) impact on stress sensitivity, with greater associations when high vs*.* low exposure levels are compared (H3); second, across groups, the impact of specific types of bullying victimization on stress sensitivity is greater in service users vs*.* controls, service users vs*.* siblings, and siblings vs*.* controls (H4). Lastly, to test whether specific bullying types modify specific forms of affective and psychotic reactivity, the following exploratory analyses were conducted: Within groups, individuals’ response to specific stressors in daily life (i.e. event-related, activity-related, social stress) is modified by specific types of bullying victimization (i.e. physical, verbal, indirect bullying), with greater associations in individuals exposed to high *vs.* low exposure levels (H5).

## Materials and methods

### Sample

Data were derived from the Youth Experience Study (YES), a study conducted to investigate candidate mechanisms involved in linking adverse childhood experiences and youth mental health. Dataset version 1.1 was used for the current analysis. This version differs from version 1.0 used in earlier work [40] in the group status used for one individual. A sample of help-seeking youth (service users) were recruited from the Mutsaers Foundation (MF) by treatment coordinators and leaflets and posters were distributed in waiting areas of all outpatient locations of MF. The MF offers secondary mental health services for young individuals in Limburg, the Netherlands. The following broad inclusion criteria were used: aged 12–20 years; currently receiving treatment from MF. Exclusion criteria were: being diagnosed with an autistic spectrum disorder according to DSM-IV with the exception of pervasive developmental disorder not otherwise specified; intellectual disability (IQ score below 70); insufficient knowledge of the Dutch language. Further, we recruited siblings of service users. Inclusion criteria were as follows: aged 12–20; participation of a biological sibling who is receiving treatment from MF. Exclusion criteria were the same as for service users with the addition of a lifetime history of receiving treatment from a mental health service. Lastly, a control sample of non-help-seeking individuals was recruited through schools from the same catchment area as MF mental health services. These schools were asked for permission to conduct the study and a letter accompanied by a leaflet was sent to parents, asking them whether their child is allowed to participate in the study. The YES was also introduced in form of an information session in class. Inclusion criteria were: aged 12–20 years, attending a school in the same catchment area as MF mental health services. Exclusion criteria were the same as for siblings. If a participant was older than 18, he/she was allowed to give written informed consent without asking the parents. The study was approved by the Medical Ethics Review Committee of Maastricht University Medical Centre in Maastricht, the Netherlands (approval number: NL37420.068.11).

### Measures

#### Socio-demographic characteristics

Socio-demographic data (i.e. age, sex, ethnicity, and level of education) was collected using a socio-demographic schedule.

#### Bullying victimization

The Retrospective Bullying Questionnaire (RBQ), a 44-item self-report questionnaire [45], was used to assess bullying victimization. The questionnaire measures exposure to bullying at primary and secondary school, while the precise timing of exposure prior to assessment is not specified. Three types of bullying were assessed: physical (hit/punched, stolen property), verbal (called names, threatened), and indirect (spread lies, excluded) bullying. In addition to assessing exposure to bullying victimization, the RBQ asks more general questions about individuals’ experiences at school (e.g. whether individuals were happy), details about the bullying incident (e.g. the number of bullies involved, reasons individuals believe they were bullied), and also bullying experiences at the workplace. For this study, we used 2 items asking for frequency and intensity of each bullying type (physical, verbal, indirect) for primary as well as secondary school resulting in 12 items rated on a 5-point scale ranging from 1 to 5 [45]. Frequency was assessed by asking participants how often they were exposed to bullying (1 = ‘never’, 5 = ‘constantly’) and intensity was assessed by asking to evaluate the seriousness (1 = ‘not at all’, 5 = ‘extremely serious’). For primary hypotheses, sum scores were calculated by adding items assessing the frequency and intensity of bullying experiences (12 items; range sum score, 12–60, Cronbach’s alpha, *α* = 0.90) and, for secondary and exploratory hypotheses, sum scores were calculated for three specific bullying types (4 items; range sum score 4–20; physical bulling, *α* = 0.77; verbal bullying, *α* = 0.84; indirect bullying, *α* = 0.87), respectively. Good psychometric properties have been reported for this measure [45].

#### Depressive, anxiety, and psychotic symptoms

The Beck Depression Inventory (BDI-II), a well-established questionnaire consisting of 21 items, was completed to assess depressive symptoms over the past 2 weeks (4-point scale ranging from 0 to 3). A Dutch version of the State-Trait Anxiety Inventory (STAI) was used to assess state and trait anxiety. The first part (STAI-DY1) measures trait (20 items) and the second part (STAI-DY2) assesses state (40 items) anxiety, both rated on a 4-point scale (ranging from 1 to 4; 1 = not at all, 4 = very much). The Community Assessment of Psychic Experiences (CAPE) was used to assess the frequency and distress of positive (20 items) and negative (14 items) sub-clinical psychotic and depressive (8 items) symptoms (rated on a 4-point scale ranging from 0 to 3; 0 = not at all, 3 = very much). For all measures, good psychometric properties have been [46–48] demonstrated.

#### Momentary stress, negative affect, and psychotic experiences

Momentary stress, negative affect, and psychotic experiences were assessed using the experience sampling method (ESM), an intensive self-assessment technique to assess subjective experiences and social contexts in real life, outside the research laboratory with high ecological validity [35]. A personal digital assistant (PsyMate) was used for data collection. In accordance with previous ESM studies [49, 50], the PsyMate beeped 10 times a day on 6 consecutive days at unpredictable moments between 7:30 am and 10:30 pm (scheduled at random within set time blocks of 90 min). Event-related, activity-related, and social stress were defined as unpleasant events, activities, and social situations occurring in daily life. Sufficient concurrent validity with other stress measures has been reported [51].

Momentary stress was calculated by computing the mean score of six items assessing event-related, activity-related, and social stress. Event-related stress was measured asking participants to report the pleasantness of the most important event that had happened since the last beep on a 7-point scale ranging from ‘very unpleasant’ (rating of − 3) to ‘very pleasant’ (rating of 3). To ensure that higher ratings indicate higher levels of stress and pleasant events are excluded from analyses, the item was recoded (ratings of − 3 were coded as 4, − 2 as 3, − 1 as 2, and neutral events as 1, while pleasant events were coded as 0). Activity-related stress was assessed by asking ‘What am I doing (just before the beep)’ (e.g. being at work/school, doing household, eating/drinking) and three additional items (‘I would prefer doing something else’, ‘This activity is difficult for me’, ‘I can do this well’ [reversed]) ranging from ‘not at all’ (rating of 1) to ‘very much’ (rating of 7). Social stress was measured by asking participants about their current social situation (e.g. ‘I am alone’, ‘I am with my family’, ‘I am with my friends’) and to rate this using the items ‘I find the people I am with pleasant’ [reversed] (if with someone) or ‘I like to be alone’ [reversed] (if alone) ranging from ‘not at all’ (rating of 1) to ‘very much’ (rating of 7).

Negative affect was assessed using five items asking participants to report the degree of feeling anxious, lonely, insecure, irritated, and down. Psychotic experiences were measured using eight items (‘I see things that aren’t really there’, ‘I hear things that aren’t really there’, ‘I feel suspicious/paranoid’, ‘I feel harried’, ‘I feel unreal’, ‘My thoughts are influenced by other’, ‘I can’t get these thoughts out of my head’, ‘I feel like I am losing control’). All items were rated on a 7-point scale (1 = ‘not at all’, 7 = ‘very much’) and mean scores were calculated to compute both variables. High levels of internal consistency and good concurrent validity with interviewer-rated measures has been reported [38].

### Statistical analysis

First, we compared socio-demographic characteristics and psychopathological domains (i.e. standardized BDI-II, STAI-DY1/DY2, and CAPE scores) across groups using linear regression and *χ*^2^-tests. Second, the MIXED command in Stata 15 was used to fit linear mixed models. This statistical modelling technique is needed as ESM data has a multilevel structure with multiple observations nested within participants. Maximum likelihood estimation of these models allows all available data to be used under the relatively unrestricted assumption that data is missing at random. We fitted models with momentary stress (event-related, activity-related, and social stress; primary and secondary hypotheses [H1-H4]: overall mean score including all stress items; exploratory analyses [H5]: mean score of specific stressors) as the continuous independent variable and (i) negative affect and (ii) psychotic experiences as the outcome variable, while controlling for potential confounders and variables associated with missing values (i.e. age, sex, ethnicity, level of education). To test whether associations between momentary stress and (i) negative affect and (ii) psychotic experiences are modified by exposure to bullying victimization at school (i.e. exposure at primary and secondary school combined; continuous total scores) and group (service users, siblings, and controls), two-way (stress × bullying, stress × group, bullying × group) and three-way (stress × bullying × group) interaction terms were simultaneously added into models. Wald tests were performed using the TESTPARM command to evaluate significance of three-way interaction terms to the model. The continuous stress and continuous bullying variables were standardized (mean = 0, S.D. = 1) for interpreting significant three-way interaction terms [52] and the LINCOM command was used to compute linear combinations of coefficients to test the hypotheses that, within each group, the association of momentary stress with (i) negative affect and (ii) psychotic experiences was greater in individuals exposed to high *vs.* those exposed to low levels of bullying victimization (± 1 S.D. of standardized continuous bullying victimization total scores; primary hypotheses [H1]: exposure to overall bullying; secondary and exploratory hypothesis [H3 and H5]: exposure to specific bullying types [53, 54]. Lastly, we investigated whether the impact of bullying victimization on stress sensitivity differed across groups by comparing the differences in the magnitude of associations of momentary stress with (i) negative affect and (ii) psychotic experiences between those exposed to high *vs*. low levels of bullying victimization (primary hypotheses [H2]: exposure to overall bullying; secondary hypotheses [H4]: exposure to specific bullying types) in service users compared to controls, service users compared to siblings, and siblings compared to controls. Separate models for momentary stress (overall as well as three specific stressors) and bullying exposure (overall as well as three specific types) were calculated, resulting in 2 models for primary hypotheses, 6 models for secondary hypotheses, and 24 models for exploratory analyses. We adjusted significance levels of Wald tests for three-way interactions to correct for Type-1 error proliferation using family-wise error-corrected p values (*p*FWE) by multiplying the unadjusted *p* value by the total number of tests (*N* = 8 for primary and secondary analyses and *N* = 24 for exploratory analyses).

## Results

### Basic sample and clinical characteristics

In total, 109 individuals were eligible to participate. Of these, 99 youths (42 service users, 17 siblings, and 40 controls) completed the ESM with ≥ 20 valid responses over the 6-day assessment period as well as the BDI-II, STAI-DY1/DY2, CAPE, and RBQ. Thus, a high proportion of those initially assessed were included in the analysis (i.e. 90.8% of 109). There were, within groups, no differences between individuals who completed ESM assessments and those who did not with regard to socio-demographic characteristics and other variables. Groups did not differ in age, sex, or ethnicity (Table 1). However, there was evidence for higher levels of depression (BDI-II: *β* = 0.71, *p* = 0.001; *B* = 1.08 *p* < 0.001), state (*β* = 0.50, *p* = 0.025; *β* = 0.59, *p* = 0.40) and trait (*β* = 0.59, *p* = 0.004; *B* = 0.95, *p* < 0.001) anxiety, and negative (*β* = 0.42, *p* = 0.057; *β* = 0.73, *p* = 0.011) and positive (*β* = 0.75, *p* < 0.001; *β* = 0.84, *p* = 0.002) psychotic-like experiences in service users *vs.* controls and service users *vs.* siblings, respectively. As shown in Table 2, service users were exposed to higher overall levels of bullying victimization compared to controls (*β* = 0.56, *p* = 0.010) and siblings (*β* = 0.60, *p* = 0.034), while, in contrast, no differences were found comparing siblings and controls (*β* = -0.03, *p* = 0.902). Further, service users reported higher levels of physical (*β* = 0.86, *p* < 0.001; *β* = 0.77, *p* = 0.005), but not verbal (*β* = 0.29, *p* = 0.188; *β* = 0.42, *p* = 0.150) and indirect (*β* = 0.37, *p* = 0.092; *β* = 0.39, *p* = 0.169) bullying compared to controls and siblings, respectively. Moreover, although not the primary aim of the current paper, it is worth mentioning that service users were more likely to report bullying-related mental health complaints, harmful behavior, and occupational problems when compared to controls and siblings (Table 2).

**Table 1:** Basic sample characteristics

	Service users (*n* = 42)	Siblings (*n* = 17)	Controls (*n* = 40)	Test statistic	*p*
Age (years), mean (S.D.)	15.4 (1.4)	15.3 (2.3)	15.6 (2.0)	*F* = 0.24, *df* = 2	0.785
Sex *n* (%)					
Female	25 (59.5)	10 (58.8)	23 (57.5)	*χ*^2^ = 0.04, *df* = 2	0.983
Male	17 (40.5)	7 (41.2)	17 (42.5)		
Ethnicity* n* (%)^a^					
White Dutch	26 (61.9)	11 (64.7)	25 (64.1)	*χ*^2^ = 0.06, *df* = 2	0.970
Other	16 (38.1)	6 (35.3)	14 (35.9)		
Level of education^b^ *n* (%)					
School	30 (71.4)	7 (41.2)	17 (42.5)	*χ*^2^ = 10.48, *df* = 2	0.033
Further	12 (28.6)	8 (47.1)	20 (50.0)		
Higher	–	2 (11.8)	3 (7.5)		
Cannabis use *n* (%)					
12-month	9 (21.4)	1 (5.9)	4 (10.0)	*χ*^2^ = 3.36, *df* = 2	0.187
Lifetime	9 (21.4)	2 (11.8)	5 (12.5)	*χ*^2^ = 1.50, *df* = 2	0.473
Attempted suicide *n* (%)					
During last year	6 (14.6)	–	–	–	–
Before age 17	8 (19.1)	–	–		
DSM-IV diagnoses *n* (%)					
Pervasive developmental disorders NOS	10 (23.8)	–	5 (12.5)	–	–
Attention-deficit and disruptive behaviour	6 (14.3)	3 (17.6)	–		
Adjustment disorders	4 (9.5)	–	–		
Anxiety disorders	2 (4.8)	–	–		
Depressive disorders	2 (4.8)	–	–		
Gender identity disorders	2 (4.8)	–	–		
Learning disorders	–	–	2 (5.0)		
Other disorders of infancy, childhood, or adolescence	5 (11.9)	–	–		
Parent–child relational problem	5 (11.9)	1 (5.9)	1 (2.5)		
Comorbid condition^c^	24 (57.1)	2 (11.8)	–		
None	6 (14.3)	13 (76.5)	32 (80.0)		
BDI-II sum sores, mean (S.D.)	12.8 (9.2)	3.9 (3.3)	6.9 (7.0)	*F* = 10.5, *df* = 2	< 0.001
CAPE sum scores, mean (S.D.)					
Positive	10.0 (9.4)	3.9 (3.2)	4.6 (3.9)	*F* = 8.28, *df* = 2	< 0.001
Negative	9.9 (6.7)	5.6 (3.8)	7.4 (4.8)	*F* = 3.88, *df* = 2	0.024
Depressive	7.7 (4.0)	4.2 (1.8)	4.7 (3.4)	*F* = 9.90, *df* = 2	< 0.001
STAI-DY1 (trait anxiety)^a^ sum scores, mean (S.D.)	35.5 (10.6)	30.2 (6.8)	31.1 (7.2)	*F* = 3.47, *df* = 2	0.035
STAI-DY2 (state anxiety)^a^ sum scores, mean (S.D.)	85.6 (20.8)	67.1 (9.2)	74.1 (16.4)	*F* = 8.12, *df* = 2	< 0.001
Number of valid beeps Mean (range, min–max)	44.16 (25–59)	43.4 (23–57)	44.9 (24–58)	*F* = 0.27, *df* = 2	0.754

	Cases vs controls^d^		Siblings vs controls^d^		Cases vs siblings^d^	
	*β* (95% CI)	*p*	*β* (95% CI)	*p*	*β* (95% CI)	*p*
BDI-II	0.71 (0.30–1.11)	0.001	− 0.37 (− 0.89 to 0.16)	0.168	1.08 (0.55–1.60)	< 0.001
CAPE						
Positive	0.75 (0.34–1.17)	< 0.001	− 0.09 (− 0.63 to 0.44)	0.735	0.84 (0.31–1.38)	0.002
Negative	0.42 (− 0.01 to 0.85)	0.057	− 0.31 (− 0.87 to 0.25)	0.269	0.73 (0.17–1.29)	0.011
Depressive	0.80 (0.40–1.21)	< 0.001	− 0.12 (− 0.64 to 0.41)	0.666	0.92 (0.39–1.45)	0.001
STAI-DY1	0.50 (0.06–0.93)	0.025	− 0.09 (− 0.65 to 0.47)	0.748	0.59 (0.03–1.14)	0.040
STAI-DY2	0.59 (0.19–0.99)	0.004	− 0.36 (− 0.88 to 0.16)	0.170	0.95 (0.43–1.46)	< 0.001

*S.D.* standard deviation, *df* degrees of freedom, *β* standardized regression coefficients (mean score differences); CI, confidence interval

^a^Missing values: ethnicity = 1, BDI = 1, STAI-DY1 = 1, STAI-DY2 = 2

^b^Categories defined as: school (primary education, LBO, MAVO, VMBO), further (MBO, HAVO, VWO), and higher (HBO, WO) of the Dutch educational system

^c^Consisting of the following diagnostic categories in the case group: Additional codes (Parent–child relational problem, 33.3%; Borderline intellectual functioning, 13.3%; Neglect of child, 6.7%), Attention-deficit and disruptive behaviour disorders (10%), Learning disorders (10%), Personality disorders (6.7%), Mild mental retardation (6.7%), Anxiety disorders (3.3%), Dissociative disorders (3.3%), Tic disorders (3.3%), Amphetamine related disorders (3.3%)

^d^Standardized mean score differences across groups

**Table 2:** Exposure to bullying victimization and related mental health complaints within and across groups

	Service users (*n* = 42)	Siblings (*n* = 17)	Controls (*n* = 40)	Service users vs*.* controls	Service users vs*.* siblings	Siblings vs*.* controls
Bullying victimization *n* (%)						
Any	32 (76.2)	11 (64.7)	27 (67.5)	OR = 1.5 (0.6–4.1)	OR = 1.7 (0.5–5.9)	OR = 0.9 (0.3–2.9)
Physical bullying	20 (47.6)	2 (11.8)	6 (15.0)	OR = 5.2 (1.8–14.8)*	OR = 6.8 (1.4–33.6)*	OR = 0.8 (0.1–4.2)
Verbal bullying	24 (57.1)	10 (58.8)	23 (57.5)	OR = 1.0 (0.4–2.4)	OR = 0.9 (0.3–2.9)	OR = 1.1 (0.3–3.3)
Indirect bullying	27 (64.3)	9 (52.9)	21 (52.5)	OR = 1.6 (0.7–3.9)	OR = 1.6 (0.5–5.0)	OR = 1.0 (0.3–3.2)
Bullying victimization, only severe and frequent ^a^ *n* (%)						
Any	21 (50.0)	2 (11.8)	10 (25.0)	OR = 3.0 (1.2–7.7)*	OR = 7.5 (1.5–37.0)*	OR = 0.4 (0.1–2.1)
Physical bullying	12 (28.6)	0 (0.0)	1 (2.5)	OR = 15.6 (1.9–126.7)*	–	–
Verbal bullying	14 (33.3)	1 (5.9)	5 (12.5)	OR = 3.5 (1.1–10.9)*	OR = 8.0 (0.9–66.6)	OR = 0.4 (0.0–4.1)
Indirect bullying	16 (38.1)	2 (11.8)	9 (22.5)	OR = 2.1 (0.8–5.6)	OR = 4.6 (0.9–22.9)	OR = 0.5 (0.1–2.4)
Bullying victimization, mean (S.D.)						
Overall	24.6 (11.3)	18.8 (7.0)	19.1 (8.0)	*β* = 0.56 (0.14–0.99)*	*β* = 0.60 (0.05–0.15)*	*β* = − 0.03 (− 0.59 to 0.52)
Physical bullying	7.2 (3.7)	4.8 (2.4)	4.6 (1.6)	*β* = 0.86 (0.46–1.26)**	*β* = 0.77 (0.24–1.29)*	*β* = 0.09 (− 0.44 to 0.62)
Verbal bullying	8.5 (4.8)	6.8 (2.8)	7.3 (3.8)	*β* = 0.29 (− 0.15 to 0.73)	*β* = 0.42 (− 0.15 to 0.98)	*β* = −0.12 (−0.70 to 0.45)
Indirect bullying	8.9 (4.9)	7.2 (3.6)	7.3 (4.1)	*β* = 0.37 (− 0.06 to 0.81)	*β* = 0.39 (− 0.17 to 0.96)	*β* = − 0.02 (− 0.59 to 0.54)
Self-perceived long-term effects	17 (54.8)	1 (9.1)	5 (18.5)	OR = 5.3 (1.6–17.8)*	OR = 12.1 (1.4–106.8)*	OR = 0.4 (0.0–4.3)
Bullying-related mental health complaints *n* (%)						
Absence school	15 (46.9)	2 (18.2)	5 (18.5)	OR = 3.9 (1.2–12.8)*	OR = 4.0 (0.7–21.4)	OR = 1.0 (0.2–6.0)
Suicidal thoughts	17 (53.1)	1 (9.1)	6 (22.2)	OR = 4.0 (1.3–12.4)*	OR = 11.3 (1.3–99.2)*	OR = 0.4 (0.0–3.3)
Vivid memories	20 (62.5)	1 (9.1)	9 (33.3)	OR = 3.3 (1.1–9.8)*	OR = 16.7 (1.9–146.9)*	OR = 0.2 (0.2–1.8)
Nightmares	9 (28.1)	0 (0.0)	3 (11.1)	OR = 3.1 (0.8–13.0)	–	–
Re-living the event	13 (40.6)	0 (0.0)	4 (14.8)	OR = 3.9 (1.1–14.1)*	–	–
Flashbacks	14 (43.8)	1 (9.1)	6 (22.2)	OR = 2.7 (0.9–8.6)	OR = 7.8 (0.9–68.2)	OR = 0.4 (0.0–3.3)
Distressed in situations	16 (50.0)	0 (0.0)	3 (11.1)	OR = 8.0 (2.0–32.0)*	–	–

*S.D*. standard deviation, *df* degrees of freedom, *β* standardized regression coefficients (mean score differences), *CI* confidence interval, *OR* odds ratio

*p < 0.05, **p < 0.001

^a^Defined as being bullied “sometimes” or more often (frequency) and the experience were evaluated as “quite serious” or “extremely serious” (intensity), for analyses all exposure levels were used

### Association between momentary stress and negative affect by bullying victimization and group

There was evidence in support of primary and secondary hypotheses that exposure to overall bullying victimization as well as physical bullying, but not verbal and indirect bullying, modified the association of momentary stress with negative affect (Table 3). Evidence for effect modification by levels of bullying exposure within and across groups was evidenced by statistically significant 3-way interaction effects described below (Table 3).

**Table 3:** Association of stress with negative affect and psychotic experiences, by levels of bullying victimization in service users, siblings, and controls

	Service users		Siblings		Controls		Wald test for interaction^c^		
	adj. *β* (95% CI)	*p*	adj. *β* (95% CI)	*p*	adj. *β* (95% CI)	*p*	*χ*^2^ (df)	*p*	*p*FWE
*Outcome: negative affect*									
Momentary stress^b^ × bullying × group									
Overall bullying exposure							12.83 (2)	0.002	0.013
High (mean + 1 SD)	0.25 (0.21–0.29)	< 0.001	0.11 (− 0.00 to 0.23)	0.054	0.11 (0.04–0.18)	0.003			
Average (mean)	0.20 (0.17–0.24)	< 0.001	0.08 (0.01–0.14)	0.018	0.16 (0.12–0.21)	< 0.001			
Low (mean − 1 SD)	0.16 (0.10–0.21)	< 0.001	0.04 (− 0.06 to 0.13)	0.412	0.22 (0.17–0.27)	< 0.001			
High vs. low^d^	0.09 (0.04–0.15)	0.002	0.07 (− 0.10 to 0.24)	0.392	-0.11 (− 0.21 to 0.01)	0.024			
Physical bullying							28.91 (2)	< 0.001	< 0.001
High (mean + 1 SD)	0.24 (0.20–0.28)	< 0.001	0.14 (0.04–0.24)	0.005	− 0.05 (− 0.15 to 0.06)	0.358			
Average (mean)	0.20 (0.16–0.24)	< 0.001	0.08 (0.02–0.15)	0.010	0.12 (0.07–0.16)	< 0.001			
Low (mean − 1 SD)	0.17 (0.11–0.23)	< 0.001	0.02 (− 0.06 to 0.10)	0.615	0.28 (0.22–0.34)	< 0.001			
High vs. low^d^	0.07 (0.02–0.13)	0.010	0.12 (− 0.01 to 0.26)	0.073	− 0.33 (− 0.47 to − 0.19)	< 0.001			
Verbal bullying							4.69 (2)	0.100	0.767
Indirect bullying							9.05 (2)	0.011	0.087
*Outcome: psychotic experiences*									
Momentary stress^b^ × bullying × group^b^									
Overall bullying exposure							10.63 (2)	0.005	0.039
High (mean + 1 SD)	0.14 (0.12–0.16)	< 0.001	0.06 (− 0.00 to 0.12)	0.051	0.04 (0.00–0.08)	0.029			
Average (mean)	0.10 (0.07–0.12)	< 0.001	0.04 (0.00–0.07)	0.036	0.05 (0.03–0.07)	< 0.001			
Low (mean− 1 SD)	0.05 (0.02–0.08)	< 0.001	0.01 (− 0.04 to 0.06)	0.667	0.06 (0.03–0.09)	< 0.001			
High vs. low^d^	0.09 (0.05–0.12)	< 0.001	0.05 (− 0.04 to 0.14)	0.278	− 0.01 (− 0.07 to 0.04)	0.575			
Physical bullying							2.47 (2)	0.291	1.0
Verbal bullying							3.46 (2)	0.178	1.0
Indirect bullying							19.35 (2)	< 0.001	< 0.001
High (mean + 1 SD)	0.15 (0.13–0.17)	< 0.001	0.07 (0.01–0.12)	0.022	0.04 (0.00–0.07)	0.038			
Average (mean)	0.10 (0.08–0.12)	< 0.001	0.04 (0.00–0.07)	0.039	0.05 (0.03–0.07)	< 0.001			
Low (mean − 1 SD)	0.05 (0.03–0.08)	< 0.001	0.00 ( −0.05 to 0.06)	0.883	0.06 (0.03–0.09)	< 0.001			
High vs. low^d^	0.10 (0.07–0.13)	< 0.001	0.06 (− 0.02 to 0.15)	0.153	− 0.03 (− 0.07 to 0.02)	0.241			

	Service users vs. controls^d^		Siblings vs. controls^d^		Service users vs. siblings^d^	
	adj. *β* (95% CI)	*p*	adj. *β* (95% CI)	*p*	adj. *β* (95% CI)	*p*
Δ high vs. low exposure levels of bullying victimization across groups						
*Outcome: negative affect*						
Momentary stress × bullying × group						
Overall bullying exposure	0.21 (0.09–0.32)	< 0.001	0.19 (− 0.01 to 0.38)	0.062	0.02 (− 0.16 to 0.20)	0.822
Physical bullying	0.40 (0.25–0.56)	< 0.001	0.45 (0.26–0.65)	< 0.001	− 0.05 (− 0.19 to 0.10)	0.497
*Outcome: psychotic experiences*						
Momentary stress × bullying × group						
Overall bullying exposure	0.10 (0.04–0.16)	0.001	0.07 (− 0.04 to 0.17)	0.222	0.04 (− 0.06 to 0.13)	0.470
Indirect bullying	0.12 (0.07–0.18)	< 0.001	0.09 (− 0.01 to 0.19)	0.070	0.03 (− 0.06 to 0.12)	0.473

*SD* standard deviation, *df* degrees of freedom, *vs.* versus, *CI* confidence interval, *adj. β* standardized regression coefficients, continuous independent variables were standardized (mean = 0, SD = 1) for interpreting significant three-way interaction terms and examining the difference in associations between high (mean + 1 SD), average (mean), and low (mean − 1 SD) levels of exposure to bullying victimization within and across groups (service users, siblings, controls), *pFWE* family-wise error-corrected *p* values were computed by multiplying the unadjusted *p* value by the total number of tests (*N* = 8) to adjust significance levels of likelihood ratio tests for three-way interactions

^a^Adjusted for age, gender, ethnicity, level of education, 12-month use of cannabis

^b^Momentary stress was calculated by combining the ratings of six items assessing event-related, activity-related, and social stress by calculating mean scores

^c^Three-way interaction as included in the following model (with *y*_ij_ for negative affect or psychotic experiences as outcome variable): *y*_ij_ = *β*_0_ + *β*_1_(STRESS_ij_) + *β*_2_(BULLYING_j_) + *β*_3_(GROUP_j_) + *β*_4_(STRESS_ij_ × BULLYING_j_) + *β*_5_(STRESS_ij_ × GROUP_j_) + *β*_6_(BULLYING_j_ × GROUP_j_) + *β*_7_(STRESS_ij_ × BULLYING_j_ × GROUP_j_) + *ε*_ij_ (full model not shown—available upon request)

^d^ Difference in the magnitude of associations of momentary stress with psychotic experiences bet ween those exposed to high vs. low levels of bullying victimization across groups (Δ high vs. low)

#### Within-group comparisons

Within groups, momentary stress was associated with higher negative affect in service users (*adj. β* = 0.09, *p* = 0.002) and lower negative affect in controls (*adj. β* = − 0.11, *p* = 0.024) when high *vs.* low overall bullying victimization levels were compared, while no differences by exposure levels were found in siblings (*adj. β* = 0.07, *p* = 0.392) (see Table 3). Analyses to test secondary hypotheses revealed that stress was associated with lower negative affect in controls comparing those with high *vs.* those with low physical bullying levels (*adj. β* = − 0.33, *p* < 0.001), whereas higher negative affect was observed in service users (*adj. β* = 0.07, *p* = 0.010) and, at trend level, siblings (*adj. β* = 0.12, *p* = 0.073). There was no evidence that verbal and indirect bullying modified the affective reactivity to stress in daily life. Results of exploratory analyses that test effect modification by levels of bullying exposure for associations of specific stressors (event-related, activity-related, and social) with negative affect are provided in Supplement 1 and Table S1.

#### Between-group comparisons

To investigate whether the impact of exposure to bullying victimization on stress sensitivity differed across groups, differences in magnitude of associations between those exposed to high *vs.* low levels of bullying victimization were examined across groups (Table 3). The difference in magnitude of associations between stress and negative affect was greater in service users than in controls when high *vs.* low levels of exposure to overall bullying victimization (*adj. β* = 0.21, *p* < 0.001) as well as physical (*adj. β* = 0.40, *p* < 0.001) bullying were compared. Further, there were differences in the magnitude of associations between stress and negative affect by physical (*adj. β* = 0.45, *p* < 0.001) and, at trend level, overall (*adj. β* = 0.19, *p* = 0.062) bullying comparing siblings *vs.* controls. No differences were found comparing service users *vs.* siblings.

### Association between stress and psychotic experiences by bullying victimization and group

There was evidence that exposure to overall bullying victimization as well as indirect bullying, but not verbal and physical bullying, amplified the association of momentary stress with psychotic experiences, as evidenced by statistically significant 3-way interaction effects described below (Tables 3).

#### Within-group comparisons

Within groups, momentary stress was associated with more intense psychotic experiences in service users (*adj. β* = 0.09, *p* < 0.001) exposed to high overall bullying victimization levels compared to those with low exposure levels, while no differences by bullying exposure were found in siblings (*adj. β* = 0.05, *p* = 0.278) and controls (*adj. β* = − 0.01, *p* = 0.575) (Table 3). Analyses of secondary hypotheses revealed that stress was associated with more intense psychotic experiences in service users (*adj. β* = 0.10, *p* < 0.001), but not in siblings (*adj. β* = 0.06, *p* = 0.153), and controls (*adj. β* = − 0.03, *p* = 0.241) comparing high *vs.* low levels of indirect bullying. There was no evidence that physical as well as verbal bullying modified the psychotic reactivity to stress in daily life. Results of exploratory analyses that test effect modification by levels of bullying exposure for associations of specific stressors (event-related, activity-related, and social) with psychotic experiences are provided in Supplement 1 and Table S2.

#### Between-group comparisons

There were differences in the magnitude of associations between momentary stress and psychotic experiences in those exposed to high *vs.* low exposure levels to overall bullying victimization comparing service users and controls (*adj. β* = 0.10, *p* = 0.001), but not service users and siblings (*adj. β* = 0.04, *p* = 0.470) and siblings and controls (*adj. β* = 0.07, *p* = 0.222) (Table 3). Further, there was evidence for differences in the magnitude of associations between stress and psychotic experiences by exposure levels to indirect bullying comparing service users and controls (*adj. β* = 0.12, *p* < 0.001), and, at trend level, siblings and controls (*adj. β* = 0.09, *p* = 0.070), but not service users and siblings (*adj. β* = 0.03, *p* = 0.473).

As groups differed considerably with regard to cannabis use, a sensitivity analysis is provided in Table S3 testing primary hypotheses while also controlling for 12-month prevalence of cannabis use. A similar pattern of findings emerged.

## Discussion

### Main findings

In line with primary and secondary hypotheses, our findings suggest that exposure to overall, as well as specific types of (i.e. physical and indirect, but not verbal), bullying victimization modifies individuals’ affective and psychotic reactivity to minor stress in daily life. While there was strong evidence that service users who were exposed to high levels of bullying victimization reported more intense (i) negative affect and (ii) psychotic experience in response to stress compared to those with low exposure levels, controls showed either no marked differences or, intriguingly, less intense negative affect (evident for physical bullying) by exposure levels. In siblings, a less consistent pattern of findings was observed.

### Methodological considerations

The current findings should be interpreted in light of potential limitations. First, bullying victimization was assessed retrospectively using a self-report measure. Thus, recall bias may have influenced reported findings [55] and recent studies also indicate that retrospectively assessed adverse childhood experiences, including bullying, may identify largely different groups of individuals [56]. However, potential effects may have been minimized due to the young age of the sample and the reduced time that had passed between exposure and assessment. Similarly, ESM measures were based on self-report. While this allows for ecologically valid assessment of experiences and contexts in real life, an important next step is to investigate how bullying victimization impacts on individuals’ stress sensitivity on other levels of investigation, including biological markers [57–59] and passively assessed sensor data [60]. Second, due to potentially high assessment burden associated with ESM assessment for some participants, selection may have influenced our findings and possibly introduced bias, particularly if differential by bullying exposure. However, studies have shown that the ESM is a feasible and reliable assessment method in adolescent and adult populations [35]. In addition, extensive briefing on the ESM procedure resulted in a sufficient number of responses (i.e. ≥ 20 valid responses with, on average, 45 observations in each group) in most participants (90.8%). Also, there was no evidence that the number of valid responses differed across groups. Thus, as maximum likelihood estimations were used allowing for use of all available data, the potential impact of selection and sampling bias are kept at a minimum. Third, while we adjusted for potential confounders (i.e. age, sex, ethnicity, level of education), other unmeasured factors could have influenced reported findings (e.g. polygenetic risk for various psychopathologies and personality traits, other socio-environmental risk factors). Further, current mental health problems may influence reporting of bullying victimization and stress sensitivity and may, as a consequence, influence the interpretation of reported findings. Time-lagged analyses would be required to adjust for levels of symptoms in analyses of experience sampling data, which, in turn, would require a higher number of observations to conduct such analyses with sufficient power. Fourth, the sibling group was comparably small (*N* = 17) and inconsistent findings may have occurred due to sampling error. Fifth, experience sampling data over the 6-day assessment period was used for cross-sectional modelling. Thus, the temporal order of stress, negative affect, and psychotic experiences were not specifically investigated. We therefore cannot rule out that reverse causality may have affected our findings. Similarly, using the RBQ and combining total scores of bullying exposure at primary and secondary school did not allow us for investigating the precise timing of the effects of bullying exposure on stress sensitivity. Thus, we cannot rule out that some participants may have been exposed to bullying during ESM assessments and hence timing of exposure, mechanism, and outcome could not be established. Future studies may investigate the effects of timing of bulling exposure on stress sensitivity using time-lagged analyses potentially in combination with multilevel moderated mediation models and a cohort design to test for temporality as an important criterion for establishing causality [61]. Sixth, we combined all stress items to test primary and secondary hypotheses. However, using a composite measure of minor stressors may require further scrutiny by psychometric experience sampling studies. Last, we decided to recode a bipolar scale assessing event-related stress from “very unpleasant” (coded as -3) to “very pleasant” (coded as 3) into a unipolar scale including only unpleasant and neutral events that have happened since the last beep (sores ranging from 1 to 4). This may have resulted in potential underrepresentation of event-related stress in the composite stress score. We computed sensitivity analyses including the bipolar event-related stress scale and found no marked differences for reported associations (see Table S4).

### Comparison with previous research

In recent years, evidence has accumulated that exposure to adverse childhood experiences, including bullying victimization [4–12], is associated with an increased risk of developing mental health problems. However, our understanding of candidate mechanisms remains limited, especially in youth. A process of sensitization that may ultimately lead to lasting changes in individuals’ responses to stress has been proposed to form a common mechanistic pathway that may partly explain associations between exposure to socio-environmental risk and psychopathology [28]. At a behavioral level, this proposition has been investigated using ESM and is largely supported by findings of an increased affective and psychotic reactivity in response to minor daily stressors in adults with various mental health problems and experiences of childhood trauma and adult life events [36–39].

In the current study, we found, for the first time, that young help-seeking individuals who were exposed to high levels of bullying victimization at elementary and/or secondary school responded with more intense negative affect and psychotic experiences to minor stress in their daily lives compared to those with low exposure levels. These findings are in accordance with reported effects of childhood trauma on stress sensitivity derived from the same sample [40] and may lend further support to behavioral sensitization as a process that emerges from adversity and that may contribute to push people along pathways to poor mental health outcomes in daily life in developmentally early stages of psychopathology.

In contrast, the response to stress was not differentially amplified by bullying exposure levels in controls. Specifically, controls exposed to high, but not low, exposure levels to physical bullying appeared to be resilient to its effects indicated by less intense negative affect in response to stress comparing high *vs.* low exposure levels. This is an interesting finding and parallels previous findings in which physical abuse and neglect [40] as well as sexual abuse [38] were found to be associated with lower negative affect in response to stress in controls. This may suggest that high levels of exposure to more intrusive forms of adversity may lead to the development of resilience towards subsequent stress in some individuals who do not develop help-seeking behavior, though some inconsistencies were observed in previous studies [38, 40] and direct replication studies are needed before firm conclusion can be drawn. We may speculate, however, that various protective factors may partly explain this finding, especially if they are differentially utilized and/or available in controls compared to service users, including good interpersonal relationships, social support, various personality characteristics, positive atmosphere at home, higher levels of neighbourhood social cohesion, high self-esteem, low rumination tendencies, and low polygenetic risk [62, 63]. While tempting, this explanation needs to be carefully tested in future studies. It also corroborates, more generally, previous research that has shown that a large proportion of individuals exposed to socio-environmental risk do show resilience to its detrimental effects on mental health [64].

In siblings, a less consistent pattern of findings was observed with no differences in individuals’ affective and psychotic reactivity to stress by exposure levels except some evidence for more intense negative affect in response to stress for those exposed to high *vs.* low levels of physical bullying. These results, however, should be interpreted with caution as the sample size was small and, therefore, findings may have occurred due to sampling error. In addition, as siblings represent an intermediate risk group, it may be speculated that only a small proportion develop a heightened sensitivity to stress while others are more resilient which leads to inconsistent findings at the group level. Notably, we found no evidence that verbal bullying modified the affective and psychotic reactivity in response to stress in all groups.

Interestingly, secondary analyses revealed that physical bullying at school was associated with more intense negative affect, whereas indirect bullying was associated with more intense psychotic experiences in response to stress in daily lives of help-seeking young individuals. These findings may be interpreted in light of cognitive models of psychosis [65, 66] in which various psychological factors, dysfunctional schemas, and cognitive biases are thought to be crucial in the development and maintenance of delusional ideations, one form of psychotic experiences. A core feature of delusions is thought to be an unfounded belief, and not a founded ‘proof’, that harm will occur from others. While speculative, indirect bullying may be more strongly associated with the development of persecutory beliefs of other people wanting to harm than other types of bullying which are more directly associated with physical violence, leading to an increased likelihood to respond with delusional ideations to daily life stressors. In following this line, one would expect that the psychotic reactivity to socially stressful situations is especially amplified by indirect bullying. This was, however, not the case in our exploratory analyses after we adjusted for multiple testing. Instead, indirect bullying was associated with an elevated psychotic reactivity to activity-related stress in service users. To the best of our knowledge, no study has specifically investigated differential associations of various bullying types with specific symptom domains of the psychosis spectrum.

Although strong evidence was found that bullying victimization modifies affective and psychotic reactivity to minor stress in young help-seeking individuals, future studies should further investigate effects of poly- and re-victimization on stress sensitivity. Arguably, exposure to various adverse childhood experiences and other socio-environmental risk factors, the so-called exposome [67], may lead to an accumulation of risk by progressively increasing individuals’ sensitivity to stress that may, in turn, contribute to the develop and maintenance of mental health problems. This approach would also account for findings that most risk factors are prone to cluster within a relatively small number of vulnerable individuals [2, 6] and tend to be associated with mental disorders in a dose–response fashion [15]. Additionally, more research is needed that focusses on investigating timing of bullying exposure, mechanism, and outcome by conducting well-controlled cohort studies to test whether elevated stress sensitivity mediates the association between exposure to and the onset of mental disorders. In using this study design, the potential buffering role of protective factors (e.g. number of close relationships, coping skills, personality traits) on stress sensitivity as well as potential complex interactions with other socio-environmental and genetic risk factors may be further investigated [62]. Lastly, in line with findings of frequently co-occurring psychopathological domains, especially at a developmentally early stage [23], we found high levels of depressive, anxiety, and psychotic symptoms and high proportions of non-specific diagnoses and comorbidity in service users. This further supports dimensional models of psychopathology [18, 19, 21] as well as notions of extended and transdiagnostic phenotypes [22].

## Conclusion

Our findings suggest that individuals’ response to minor stress in daily life may represent a putative risk or resilience mechanism through which exposure to bullying victimization may impact on mental health in youth. As dissipation of detrimental effects of bullying victimization on mental health has recently been reported to occur over time [7], programs that aim to prevent bullying victimization at school and inform teachers, parents, and the general public remain the ultimate goal. There is also a pressing need to directly assess bullying victimization in youth mental health services to integrate and directly tackle these adverse experiences in psychological interventions. Finally, to interrupt the process of prolonged sensitization to stress and alleviate individuals’ mental health burden in daily life, novel mHealth tools (e.g. ecological momentary interventions) may be used to provide treatment components in real life using interactive delivery schemes to extend psychotherapy from clinical settings to individuals’ everyday environment [35, 68, 69].

## Electronic supplementary material

Below is the link to the electronic supplementary material.

Supplementary file1 (DOCX 32 kb)

Supplementary file2 (DOCX 29 kb)

Supplementary file3 (DOCX 37 kb)

Supplementary file4 (DOCX 33 kb)

Supplementary file5 (DOCX 20 kb)
