# Supplementary material for: Bullying victimization and stress sensitivity in help-seeking youth: findings from an experience sampling study
Source: Eur Child Adolesc Psychiatry. 2020 May 13;30(4):591–605. doi: 10.1007/s00787-020-01540-5 (PMC8041697; doi:10.1007/s00787-020-01540-5)
Supplement: Supplementary file 1 — Supplementary file1 (DOCX 32 kb) [file 787_2020_1540_MOESM1_ESM.docx]

**Table S1.** Association between momentary stressors and negative affect, by levels of bullying victimization in service users, siblings, and controls^a^

|  | | | Service users | | |  | Siblings | | | |  | | Controls | | | |  | Wald test for interaction | | | | | |
| --- | --- | --- | --- | --- | --- | --- | --- | --- | --- | --- | --- | --- | --- | --- | --- | --- | --- | --- | --- | --- | --- | --- | --- |
|  | | | adj. β (95% CI) | P | |  | adj. β (95% CI) | | p | |  | | adj. β (95% CI) | | p | | χ^2^ (df) | | | p | | pFWE | |
|  | | |  |  | |  |  | |  | |  | |  | |  | |  |  | | | |  | |
| Outcome: negative affect | | | | | | | | | | | | | | | | | | | | |  |  |  |
| Event-related stress × bullying × group^b^ | | |  | |  |  |  |  | |  | |  | |  | |  | | |  | | | |  |
|  | Overall exposure to bullying | |  | |  |  |  |  | |  | |  | |  | | 0.13 (2) | | | 0.936 | | | | 1 |
|  | Physical bullying | |  | |  |  |  |  | |  | |  | |  | | 2.29 (2) | | | 0.319 | | | | 1 |
|  | Verbal bullying | |  | |  |  |  |  | |  | |  | |  | | 1.95 (2) | | | 0.378 | | | | 1 |
|  | Indirect bullying | |  | |  |  |  |  | |  | |  | |  | | 1.13 (2) | | | 0.568 | | | | 1 |
|  |  |  |  | |  |  |  |  | |  | |  | |  | |  | | |  | | | |  |
| Activity-related stress × bullying × group^b^ | | |  | |  |  |  |  | |  | |  | |  | |  | | |  | | | |  |
|  | overall exposure to bullying | |  | |  |  |  |  | |  | |  | |  | | 10.53 (2) | | | 0.005 | | | | 0.124 |
|  | Physical bullying | |  | |  |  |  |  | |  | |  | |  | | 18.35 (2) | | | <0.001 | | | | 0.002 |
|  |  | High (mean+1 SD) | 0.20 (0.16 – 0.24) | | <0.001 |  | 0.06 (-0.04 – 0.16) | 0.250 | |  | | -0.06 (-0.16 – 0.05) | | 0.290 | |  | | |  | | | |  |
|  |  | Average (mean) | 0.17 (0.13 – 0.21) | | <0.001 |  | 0.04 (-0.02 – 0.11) | 0.194 | |  | | 0.09 (0.04 – 0.13) | | <0.001 | |  | | |  | | | |  |
|  |  | Low (mean-1 SD) | 0.15 (0.09 – 0.21) | | <0.001 |  | 0.03 (-0.06 – 0.11) | 0.564 | |  | | 0.23 (0.17 – 0.29) | | <0.001 | |  | | |  | | | |  |
|  |  | High v. low^c^ | 0.05 (-0.01 – 0.10) | | 0.120 |  | 0.04 (-0.10 – 0.18) | 0.619 | |  | | -0.28 (-0.42 - -0.14) | | <0.001 | |  | | |  | | | |  |
|  | Verbal bullying | |  | |  |  |  |  | |  | |  | |  | | 7.08 (2) | | | 0.029 | | | | 0.696 |
|  | Indirect bullying | |  | |  |  |  |  | |  | |  | |  | | 6.64 (2) | | | 0.036 | | | | 0.867 |
|  |  |  |  | |  |  |  |  | |  | |  | |  | |  | | |  | | | |  |
| Social stress × bullying × group^b^ | | |  | |  |  |  |  | |  | |  | |  | |  | | |  | | | |  |
|  | Overall exposure to bullying | |  | |  |  |  |  | |  | |  | |  | | 4.62 (2) | | | 0.100 | | | | 1 |
|  | Physical bullying | |  | |  |  |  |  | |  | |  | |  | | 13.66 (2) | | | 0.001 | | | | 0.026 |
|  |  | High (mean+1 SD) | 0.13 (0.09 – 0.16) | | <0.001 |  | 0.11 (0.01 – 0.20) | 0.024 | |  | | -0.10 (-0.24 – 0.04) | | 0.147 | |  | | |  | | | |  |
|  |  | Average (mean) | 0.09 (0.05 – 0.12) | | <0.001 |  | 0.05 (-0.01 – 0.11) | 0.118 | |  | | 0.03 (-0.03 – 0.09) | | 0.283 | |  | | |  | | | |  |
|  |  | Low (mean-1 SD) | 0.05 (-0.01 – 0.10) | | 0.095 |  | -0.01 (-0.09 – 0.06) | 0.709 | |  | | 0.16 (0.10 – 0.23) | | <0.001 | |  | | |  | | | |  |
|  |  | High v. low^c^ | 0.08 (0.03 – 0.13) | | 0.004 |  | 0.12 (-0.00 – 0.25) | 0.504 | |  | | -0.26 (-0.44 - -0.08) | | 0.004 | |  | | |  | | | |  |
|  | Verbal bullying | |  | |  |  |  |  | |  | |  | |  | | 0.59 (2) | | | 0.744 | | | | 1 |
|  | Indirect bullying | |  | |  |  |  |  | |  | |  | |  | | 3.39 (2) | | | 0.183 | | | | 1 |
|  |  |  |  | |  |  |  |  | |  | |  | |  | |  | | |  | | | |  |

*Note:* SD, standard deviation; df, degrees of freedom; v., versus; CI, confidence interval; adj. β, standardized regression coefficients, continuous independent variables were standardized (mean=0, SD=1) for interpreting significant three-way interaction terms and examining the difference in associations between high (mean + 1 SD), average (mean), and low (mean – 1 SD) levels of exposure to bullying victimization within and across groups (service users, siblings, controls); *p*FWE , family-wise error-corrected p values were computed by multiplying the unadjusted p value by the total number of tests (*N*=24) to adjust signiﬁcance levels of likelihood ratio tests for three-way interactions.

^a^ Adjusted for age, gender, ethnicity, and level of education

^b^ Three-way interaction as included in the following model (with y_ij_ for negative affect as outcome variable): y_ij_  = β_0_ + β_1_(STRESS_ij_) + β_2_(BULLYING _j_) + β_3_(GROUP_j_) + β_4_(STRESS_ij_ × BULLYING _j_) + β_5_(STRESS_ij_ × GROUP_j_) + β_6_(BULLYING _j_ × GROUP_j_) + β_7_(STRESS_ij_ × BULLYING _j_ × GROUP_j_) + ε_ij_ (full model not shown - available upon request)

^c^ Difference in the magnitude of associations of momentary stress with negative affect between those exposed to high v. low levels of bullying victimization across groups (Δ high v. low):

|  | | Service users *vs.* controls |  |  | Siblings *vs.* controls |  |  | Service users *vs.* siblings | |  |  |
| --- | --- | --- | --- | --- | --- | --- | --- | --- | --- | --- | --- |
|  | | adj. β (95% CI) | p |  | adj. β (95% CI) | P |  | adj. β (95% CI) | | p |  |
|  | | Outcome: negative affect | | | | | | | | | |
| Δ high vs. low exposure levels  of bullying victimization across groups | |  |  |  |  |  |  | |  |  |  |
| Activity-related stress × bullying × group | |  |  |  |  |  |  | |  |  |  |
|  | Physical bullying | 0.33 (0.18 – 0.48) | <0.001 |  | 0.32 (0.12 – 0.52) | 0.002 |  | | 0.01 (-0.14 – 0.16) | 0.891 |  |
| Social stress × bullying × group | |  |  |  |  |  |  | |  |  |  |
|  | Physical bullying | 0.34 (0.15 – 0.53) | <0.001 |  | 0.39 (0.17 – 0.61) | 0.001 |  | | -0.05 (-0.18 – 0.09) | 0.522 |  |
|  |  |  |  |  |  |  |  | |  |  |  |
