# Supplementary material for: Bullying victimization and stress sensitivity in help-seeking youth: findings from an experience sampling study
Source: Eur Child Adolesc Psychiatry. 2020 May 13;30(4):591–605. doi: 10.1007/s00787-020-01540-5 (PMC8041697; doi:10.1007/s00787-020-01540-5)
Supplement: Supplementary file 2 — Supplementary file2 (DOCX 29 kb) [file 787_2020_1540_MOESM2_ESM.docx]

**Table S2.** Association between momentary stressors and psychotic experiences, by levels of bullying victimization in service users, siblings, and controls^a^

|  | | | Service users | | |  | Siblings | | | |  | | Controls | | | |  | |  | Wald test for interaction | | | | | |  |
| --- | --- | --- | --- | --- | --- | --- | --- | --- | --- | --- | --- | --- | --- | --- | --- | --- | --- | --- | --- | --- | --- | --- | --- | --- | --- | --- |
|  | | | adj. β (95% CI) | P | |  | adj. β (95% CI) | | p | |  | | adj. β (95% CI) | | p | |  | | χ^2^ (df) | | | p | | pFWE | |  |
|  | | |  |  | |  |  | |  | |  | |  | |  | |  | |  | | |  | | | |  |
| Outcome: psychotic experiences | | | | | | | | | | | | | | | | | | | | | | |  | | |  |
| Event-related stress × bullying × group^b^ | | |  | |  |  |  |  | |  | |  | |  | |  | |  | | |  | | | |  |  |
|  | Overall exposure to bullying | |  | |  |  |  |  | |  | |  | |  | |  | | 3.01 (2) | | | 0.222 | | | | 1 |  |
|  | Physical bullying | |  | |  |  |  |  | |  | |  | |  | |  | | 11.42 (2) | | | 0.003 | | | | 0.080 |  |
|  | Verbal bullying | |  | |  |  |  |  | |  | |  | |  | |  | | 0.21 (2) | | | 0.902 | | | | 1 |  |
|  | Indirect bullying | |  | |  |  |  |  | |  | |  | |  | |  | | 5.12 (2) | | | 0.077 | | | | 1 |  |
|  |  |  |  | |  |  |  |  | |  | |  | |  | |  | |  | | |  | | | |  |  |
| Activity-related stress × bullying × group^b^ | | |  | |  |  |  |  | |  | |  | |  | |  | |  | | |  | | | |  |  |
|  | Overall exposure to bullying | |  | |  |  |  |  | |  | |  | |  | |  | | 6.37 (2) | | | 0.041 | | | | 1 |  |
|  | Physical bullying | |  | |  |  |  |  | |  | |  | |  | |  | | 1.17 (2) | | | 0.557 | | | | 1 |  |
|  | Verbal bullying | |  | |  |  |  |  | |  | |  | |  | |  | | 2.07 (2) | | | 0.355 | | | | 1 |  |
|  | Indirect bullying | |  | |  |  |  |  | |  | |  | |  | |  | | 13.27 (2) | | | 0.001 | | | | 0.032 |  |
|  |  | High (mean+1 SD) | 0.13 (0.10 – 0.15) | | <0.001 |  | 0.04 (-0.01 – 0.10) | 0.131 | |  | | 0.03 (-0.00 – 0.07) | | 0.085 | |  | |  | | |  | | | |  |  |
|  |  | Average (mean) | 0.09 (0.07 – 0.11) | | <0.001 |  | 0.03 (-0.01 – 0.06) | 0.151 | |  | | 0.05 (0.02 – 0.07) | | <0.001 | |  | |  | | |  | | | |  |  |
|  |  | Low (mean-1 SD) | 0.04 (0.01 – 0.07) | | 0.004 |  | 0.01 (-0.05 – 0.06) | 0.803 | |  | | 0.06 (0.03 – 0.09) | | <0.001 | |  | |  | | |  | | | |  |  |
|  |  | High v. low^c^ | 0.09 (0.06 – 0.12) | | <0.001 |  | 0.04 (-0.05 – 0.12) | 0.417 | |  | | -0.02 (-0.07 – 0.03) | | 0.367 | |  | |  | | |  | | | |  |  |
|  |  |  |  | |  |  |  |  | |  | |  | |  | |  | |  | | |  | | | |  |  |
| Social stress × bullying × group^b^ | | |  | |  |  |  |  | |  | |  | |  | |  | |  | | |  | | | |  |  |
|  | Overall exposure to bullying | |  | |  |  |  |  | |  | |  | |  | |  | | 3.97 (2) | | | 0.140 | | | | 1 |  |
|  | Physical bullying | |  | |  |  |  |  | |  | |  | |  | |  | | 0.90 (2) | | | 0.634 | | | | 1 |  |
|  | Verbal bullying | |  | |  |  |  |  | |  | |  | |  | |  | | 2.57 (2) | | | 0.280 | | | | 1 |  |
|  | Indirect bullying | |  | |  |  |  |  | |  | |  | |  | |  | | 6.45 (2) | | | 0.040 | | | | 0.952 |  |
|  |  |  |  | |  |  |  |  | |  | |  | |  | |  | |  | | |  | | | |  |  |

*Note:* SD, standard deviation; df, degrees of freedom; v., versus; CI, confidence interval; adj. β, standardized regression coefficients, continuous independent variables were standardized (mean=0, SD=1) for interpreting significant three-way interaction terms and examining the difference in associations between high (mean + 1 SD), average (mean), and low (mean – 1 SD) levels of exposure to bullying victimization within and across groups (service users, siblings, controls); *p*FWE , family-wise error-corrected p values were computed by multiplying the unadjusted p value by the total number of tests (*N*=24) to adjust signiﬁcance levels of likelihood ratio tests for three-way interactions.

^a^ Adjusted for age, gender, ethnicity, level of education, and childhood trauma

^b^ Three-way interaction as included in the following model (with y_ij_ for psychotic experiences as outcome variable): y_ij_  = β_0_ + β_1_(STRESS_ij_) + β_2_(BULLYING_j_) + β_3_(GROUP_j_) + β_4_(STRESS_ij_ × BULLYING_j_) + β_5_(STRESS_ij_ × GROUP_j_) + β_6_(BULLYING_j_ × GROUP_j_) + β_7_(STRESS_ij_ × BULLYING_j_ × GROUP_j_) + ε_ij_ (full model not shown - available upon request)

^c^ Difference in the magnitude of associations of momentary stress with psychotic experiences between those exposed to high v. low levels of bullying victimization across groups (Δ high v. low):

|  | | *Cases vs. controls* | |  | *Siblings vs. controls* | |  | *Cases vs. siblings* | | | |  |
| --- | --- | --- | --- | --- | --- | --- | --- | --- | --- | --- | --- | --- |
|  | | adj. β (95% CI) | p |  | adj. β (95% CI) | P |  | | adj. β (95% CI) | | p |  |
|  | |  |  |  |  |  |  |  | | |  |  |
|  | | Outcome: psychotic experiences | | | | | | | | | | |
| Δ high vs. low exposure levels  of bullying victimization across groups | |  |  |  |  |  |  | | |  |  |  |
| Activity-related stress × bullying × group | |  |  |  |  |  |  | | |  |  |  |
|  | Indirect bullying | 0.11 (0.05 – 0.17) | <0.001 |  | 0.06 (-0.04 – 0.16) | 0.249 |  | | | 0.05 (-0.04 – 0.15) | 0.280 |  |
