# Supplementary material for: Bullying victimization and stress sensitivity in help-seeking youth: findings from an experience sampling study
Source: Eur Child Adolesc Psychiatry. 2020 May 13;30(4):591–605. doi: 10.1007/s00787-020-01540-5 (PMC8041697; doi:10.1007/s00787-020-01540-5)
Supplement: Supplementary file 3 — Supplementary file3 (DOCX 37 kb) [file 787_2020_1540_MOESM3_ESM.docx]

**Table S3.** Sensitivity analysis: Association of stress with negative affect and psychotic experiences, by levels of bullying victimization in service users, siblings, and controls, and also adjusted for cannabis use^a^

|  | | | Service users | | |  | Siblings | | | |  | | Controls | | | |  | | Wald test for interaction ^c^ | | | | | | |  | |
| --- | --- | --- | --- | --- | --- | --- | --- | --- | --- | --- | --- | --- | --- | --- | --- | --- | --- | --- | --- | --- | --- | --- | --- | --- | --- | --- | --- |
|  | | | adj. β (95% CI) | P | |  | adj. β (95% CI) | | p | |  | | adj. β (95% CI) | | p | |  | | χ^2^ (df) | | p | | *p*FWE | | |  | |
|  | | |  |  | |  |  | |  | |  | |  | |  | |  | |  | |  | |  | | |  | |
| Outcome: negative affect | | | | | | | | | | | | | | | | | | | | | |  | |  |  |  |  |
| Momentary stress^b^ × bullying × group | | |  | |  |  |  |  | |  | |  | |  | |  | |  | |  | | |  | | |  |  |
|  | Overall bullying exposure | |  | |  |  |  |  | |  | |  | |  | |  | | 11.02 (2) | | 0.004 | | | 0.031 | | |  |  |
|  |  | High (mean+1 SD) | 0.26 (0.22 – 0.30) | | <0.001 |  | 0.12 (0.01 – 0.24) | 0.035 | |  | | 0.13 (0.06 – 0.20) | | <0.001 | |  | |  | |  | | |  | | |  |  |
|  |  | Average (mean) | 0.21 (0.17 – 0.25) | | <0.001 |  | 0.08 (0.02 – 0.15) | 0.009 | |  | | 0.17 (0.13 – 0.22) | | <0.001 | |  | |  | |  | | |  | | |  |  |
|  |  | Low (mean-1 SD) | 0.16 (0.11 – 0.22) | | <0.001 |  | 0.04 (-0.05 – 0.14) | 0.350 | |  | | 0.22 (0.17 – 0.27) | | <0.001 | |  | |  | |  | | |  | | |  |  |
|  |  | High v. low^d^ | 0.10 (0.04 – 0.15) | | 0.001 |  | 0.08 (-0.09 – 0.24) | 0.353 | |  | | -0.09 (-0.19 – 0.00) | | 0.061 | |  | |  | |  | | |  | | |  |  |
|  | Physical bullying | |  | |  |  |  |  | |  | |  | |  | |  | | 29.62 (2) | | <0.001 | | | <0.001 | | |  |  |
|  |  | High (mean+1 SD) | 0.25 (0.21 – 0.29) | | <0.001 |  | 0.15 (0.06 – 0.25) | 0.002 | |  | | -0.04 (-0.15 – 0.06) | | 0.443 | |  | |  | |  | | |  | | |  |  |
|  |  | Average (mean) | 0.21 (0.17 – 0.25) | | <0.001 |  | 0.09 (0.03 – 0.15) | 0.005 | |  | | 0.12 (0.08 – 0.17) | | <0.001 | |  | |  | |  | | |  | | |  |  |
|  |  | Low (mean-1 SD) | 0.17 (0.12 – 0.23) | | <0.001 |  | 0.02 (-0.06 – 0.11) | 0.562 | |  | | 0.29 (0.23 – 0.35) | | <0.001 | |  | |  | |  | | |  | | |  |  |
|  |  | High v. low^d^ | 0.07 (0.02 – 0.13) | | 0.006 |  | 0.13 (-0.00 – 0.26) | 0.055 | |  | | -0.33 (-0.47 – -0.19) | | <0.001 | |  | |  | |  | | |  | | |  |  |
|  | Verbal bullying | |  | |  |  |  |  | |  | |  | |  | |  | | 3.67 (2) | | 0.159 | | | 1.0 | | |  |  |
|  | Indirect bullying | |  | |  |  |  |  | |  | |  | |  | |  | | 7.51 (2) | | 0.023 | | | 0.175 | | |  |  |
|  |  |  |  | |  |  |  |  | |  | |  | |  | |  | |  | |  | | |  | | |  |  |
|  | Outcome: psychotic experiences | | | | | | | | | | | | | | | | | | | | | |  | | |  |  |
|  |  |  |  | |  |  |  |  | |  | |  | |  | |  | |  | |  | | |  | | |  |  |
|  | Momentary stress^b^ × bullying × group^b^ | |  | |  |  |  |  | |  | |  | |  | |  | |  | |  | | |  | | |  |  |
|  | Overall bullying exposure | |  | |  |  |  |  | |  | |  | |  | |  | | 9.87 (2) | | 0.007 | | | 0.057 | | |  |  |
|  |  | High (mean+1 SD) | 0.14 (0.12 – 0.16) | | <0.001 |  | 0.06 (-0.01 – 0.12) | 0.066 | |  | | 0.05 (0.01 – 0.08) | | 0.018 | |  | |  | |  | | |  | | |  |  |
|  |  | Average (mean) | 0.10 (0.08 – 0.12) | | <0.001 |  | 0.03 (0.00 – 0.07) | 0.046 | |  | | 0.05 (0.03 – 0.07) | | <0.001 | |  | |  | |  | | |  | | |  |  |
|  |  | Low (mean-1 SD) | 0.05 (0.02 – 0.08) | | <0.001 |  | 0.01 (-0.04 – 0.06) | 0.663 | |  | | 0.05 (0.03 – 0.08) | | <0.001 | |  | |  | |  | | |  | | |  |  |
|  |  | High v. low^d^ | 0.09 (0.05 – 0.12) | | <0.001 |  | 0.05 (-0.04 – 0.14) | 0.307 | |  | | -0.01 (-0.06 – 0.04) | | 0.707 | |  | |  | |  | | |  | | |  |  |
|  | Physical bullying | |  | |  |  |  |  | |  | |  | |  | |  | | 3.38 (2) | | 0.185 | | | 1.0 | | |  |  |
|  | Verbal bullying | |  | |  |  |  |  | |  | |  | |  | |  | | 2.97 (2) | | 0.226 | | | 1.0 | | |  |  |
|  | Indirect bullying | |  | |  |  |  |  | |  | |  | |  | |  | | 17.43 (2) | | <0.001 | | | 0.001 | | |  |  |
|  |  | High (mean+1 SD) | 0.15 (0.13 – 0.17) | | <0.001 |  | 0.07 (0.01 – 0.12) | 0.024 | |  | | 0.04 (0.01 – 0.07) | | 0.021 | |  | |  | |  | | |  | | |  |  |
|  |  | Average (mean) | 0.10 (0.08 – 0.12) | | <0.001 |  | 0.03 (0.00 – 0.07) | 0.046 | |  | | 0.05 (0.03 – 0.07) | | <0.001 | |  | |  | |  | | |  | | |  |  |
|  |  | Low (mean-1 SD) | 0.06 (0.03 – 0.08) | | <0.001 |  | 0.00 (-0.05 – 0.05) | 0.938 | |  | | 0.06 (0.03 – 0.09) | | <0.001 | |  | |  | |  | | |  | | |  |  |
|  |  | High v. low^d^ | 0.09 (0.06 – 0.12) | | <0.001 |  | 0.06 (-0.02 – 0.15) | 0.144 | |  | | -0.02 (-0.07 – 0.02) | | 0.352 | |  | |  | |  | | |  | | |  |  |
|  |  |  |  | |  |  |  |  | |  | |  | |  | |  | |  | |  | | |  | | |  |  |

*Note:* SD, standard deviation; df, degrees of freedom; v., versus; CI, confidence interval; adj. β, standardized regression coefficients, continuous independent variables were standardized (mean=0, SD=1) for interpreting significant three-way interaction terms and examining the difference in associations between high (mean + 1 SD), average (mean), and low (mean – 1 SD) levels of exposure to bullying victimization within and across groups (service users, siblings, controls); *p*FWE , family-wise error-corrected p values were computed by multiplying the unadjusted p value by the total number of tests (*N*=8) to adjust signiﬁcance levels of likelihood ratio tests for three-way interactions.

^a^ Adjusted for age, gender, ethnicity, level of education, 12-month use of cannabis

^b^ Momentary stress was calculated by combining the ratings of six items assessing event-related, activity-related, and social stress by calculating mean scores

^c^ Three-way interaction as included in the following model (with y_ij_ for negative affect or psychotic experiences as outcome variable): y_ij_  = β_0_ + β_1_(STRESS_ij_) + β_2_(BULLYING_j_) + β_3_(GROUP_j_) + β_4_(STRESS_ij_ × BULLYING_j_) + β_5_(STRESS_ij_ × GROUP_j_) + β_6_(BULLYING_j_ × GROUP_j_) + β_7_(STRESS_ij_ × BULLYING_j_ × GROUP_j_) + ε_ij_ (full model not shown - available upon request)

| **Table S3.** Sensitivity analysis: Association of stress with negative affect and psychotic experiences, by levels of bullying victimization in service users, siblings, and controls, and also adjusted for cannabis use^a^ | | | | | | | | | | | |  |
| --- | --- | --- | --- | --- | --- | --- | --- | --- | --- | --- | --- | --- |
| ^d^ Difference in the magnitude of associations of momentary stress with psychotic experiences between those exposed to high v. low levels of bullying victimization across groups (Δ high v. low): | | | | | | | | | | | |  |
|  | | *Service users vs. controls* | |  | *Siblings vs. controls* | |  | *Service users vs. siblings* | | | |  |
|  | | adj. β (95% CI) | p |  | adj. β (95% CI) | P |  | | adj. β (95% CI) | | p |  |
|  | |  |  |  |  |  |  |  | | |  |  |
| Δ high vs. low exposure levels  of bullying victimization across groups | |  | | | | | | | | | | |
|  | | Outcome: negative affect | | | | | | | | | | |
|  | |  |  |  |  |  |  | | |  |  |  |
| Momentary stress x bullying x group | |  |  |  |  |  |  | | |  |  |  |
|  | Overall bullying exposure | 0.19 (0.08 – 0.30) | 0.001 |  | 0.17 (-0.02 – 0.36) | 0.082 |  | | | 0.02 (-0.16 – 0.19) | 0.847 |  |
|  | Physical bullying | 0.40 (0.25 – 0.55) | <0.001 |  | 0.46 (0.26 – 0.65) | <0.001 |  | | | -0.06 (-0.20 – 0.09) | 0.449 |  |
|  |  |  |  |  |  |  |  | | |  |  |  |
| Outcome: psychotic experiences | | | | | | | | | | | |  |
|  | |  |  |  |  |  |  | | |  |  |  |
| Momentary stress x bullying x group | |  |  |  |  |  |  | | |  |  |  |
|  | Overall bullying exposure | 0.10 (0.04 – 0.15) | 0.002 |  | 0.06 (-0.05 – 0.16) | 0.283 |  | | | 0.04 (-0.06 – 0.13) | 0.417 |  |
|  | Indirect bullying | 0.11 (0.06 – 0.17) | <0.001 |  | 0.08 (-0.01 – 0.18) | 0.084 |  | | | 0.03 (-0.06 – 0.12) | 0.509 |  |
|  |  |  |  |  |  |  |  | | |  |  |  |
