# Supplementary material for: Bullying victimization and stress sensitivity in help-seeking youth: findings from an experience sampling study
Source: Eur Child Adolesc Psychiatry. 2020 May 13;30(4):591–605. doi: 10.1007/s00787-020-01540-5 (PMC8041697; doi:10.1007/s00787-020-01540-5)
Supplement: Supplementary file 4 — Supplementary file4 (DOCX 33 kb) [file 787_2020_1540_MOESM4_ESM.docx]

**Table S4.** Sensitivity analysis: Association of stress with negative affect and psychotic experiences, by levels of bullying victimization in service users, siblings, and controls
with bipolar event-related stress item^a^

|  | | | Service users | | | | |  | | Siblings | | | | | |  | | | Controls | | | | | |  | | | Wald test for interaction ^c^ | | | | | | | | | |  | | |  |
| --- | --- | --- | --- | --- | --- | --- | --- | --- | --- | --- | --- | --- | --- | --- | --- | --- | --- | --- | --- | --- | --- | --- | --- | --- | --- | --- | --- | --- | --- | --- | --- | --- | --- | --- | --- | --- | --- | --- | --- | --- | --- |
|  | | | adj. β (95% CI) | | P | | |  | | adj. β (95% CI) | | | p | | |  | | | adj. β (95% CI) | | | p | | |  | | | χ^2^ (df) | | | p | | | *p*FWE | | | |  | | |  |
|  | | |  | |  | | |  | |  | | |  | | |  | | |  | | |  | | |  | | |  | | |  | | |  | | | |  | | |  |
| Outcome: negative affect | | | | | | | | | | | | | | | | | | | | | | | | | | | | | | | | |  | |  | |  |  |  |  |  |
| Momentary stress^b^ × bullying × group | | |  | | |  | |  | |  | |  | | |  | | |  | | |  | | |  | | |  | | |  | | | |  | | | |  | |  |  |
|  | Overall bullying exposure | | |  | | |  | |  | |  | | |  | | |  | | |  | | |  | | |  | | | 11.10 (2) | | | 0.004 | | | | 0.031 | | |  | | |
|  |  | High (mean+1 SD) | | 0.26 (0.22 – 0.30) | | | <0.001 | |  | | 0.12 (0.01 – 0.24) | | | 0.035 | | |  | | | 0.13 (0.06 – 0.20) | | | <0.001 | | |  | | |  | | |  | | | |  | | |  | | |
|  |  | Average (mean) | | 0.21 (0.17 – 0.25) | | | <0.001 | |  | | 0.08 (0.02 – 0.15) | | | 0.009 | | |  | | | 0.17 (0.13 – 0.22) | | | <0.001 | | |  | | |  | | |  | | | |  | | |  | | |
|  |  | Low (mean-1 SD) | | 0.16 (0.11 – 0.22) | | | <0.001 | |  | | 0.04 (-0.05 – 0.14) | | | 0.350 | | |  | | | 0.22 (0.16 – 0.27) | | | <0.001 | | |  | | |  | | |  | | | |  | | |  | | |
|  |  | High v. low^d^ | | 0.10 (0.04 – 0.15) | | | 0.001 | |  | | 0.08 (-0.09 – 0.24) | | | 0.353 | | |  | | | -0.09 (-0.19 – 0.00) | | | 0.062 | | |  | | |  | | |  | | | |  | | |  | | |
|  | Physical bullying | | |  | | |  | |  | |  | | |  | | |  | | |  | | |  | | |  | | | 29.70 (2) | | | <0.001 | | | | <0.001 | | |  | | |
|  |  | High (mean+1 SD) | | 0.25 (0.21 – 0.29) | | | <0.001 | |  | | 0.15 (0.06 – 0.25) | | | 0.002 | | |  | | | -0.04 (-0.15 – 0.06) | | | 0.443 | | |  | | |  | | |  | | | |  | | |  | | |
|  |  | Average (mean) | | 0.21 (0.17 – 0.25) | | | <0.001 | |  | | 0.09 (0.03 – 0.15) | | | 0.005 | | |  | | | 0.12 (0.08 – 0.17) | | | <0.001 | | |  | | |  | | |  | | | |  | | |  | | |
|  |  | Low (mean-1 SD) | | 0.17 (0.12 – 0.23) | | | <0.001 | |  | | 0.02 (-0.06 – 0.11) | | | 0.563 | | |  | | | 0.29 (0.23 – 0.35) | | | <0.001 | | |  | | |  | | |  | | | |  | | |  | | |
|  |  | High v. low^d^ | | 0.08 (0.02 – 0.13) | | | 0.005 | |  | | 0.13 (-0.00 – 0.26) | | | 0.055 | | |  | | | -0.33 (-0.47 – -0.19) | | | <0.001 | | |  | | |  | | |  | | | |  | | |  | | |
|  | Verbal bullying | | |  | | |  | |  | |  | | |  | | |  | | |  | | |  | | |  | | | 3.67 (2) | | | 0.159 | | | | 1.0 | | |  | | |
|  | Indirect bullying | | |  | | |  | |  | |  | | |  | | |  | | |  | | |  | | |  | | | 7.61 (2) | | | 0.022 | | | | 0.178 | | |  | | |
|  |  |  | |  | | |  | |  | |  | | |  | | |  | | |  | | |  | | |  | | |  | | |  | | | |  | | |  | | |
|  | Outcome: psychotic experiences | | | | | | | | | | | | | | | | | | | | | | | | | | | | | | | | | | |  | | |  | | |
|  |  |  | |  | | |  | |  | |  | | |  | | |  | | |  | | |  | | |  | | |  | | |  | | | |  | | |  | | |
|  | Momentary stress^b^ × bullying × group | | |  | | |  | |  | |  | | |  | | |  | | |  | | |  | | |  | | |  | | |  | | | |  | | |  | | |
|  | Overall bullying exposure | | |  | | |  | |  | |  | | |  | | |  | | |  | | |  | | |  | | | 9.99 (2) | | | 0.007 | | | | 0.054 | | |  | | |
|  |  | High (mean+1 SD) | | 0.14 (0.12 – 0.16) | | | <0.001 | |  | | 0.06 (-0.01 – 0.12) | | | 0.066 | | |  | | | 0.04 (0.01 – 0.08) | | | 0.019 | | |  | | |  | | |  | | | |  | | |  | | |
|  |  | Average (mean) | | 0.10 (0.08 – 0.12) | | | <0.001 | |  | | 0.03 (0.00 – 0.07) | | | 0.046 | | |  | | | 0.05 (0.03 – 0.07) | | | <0.001 | | |  | | |  | | |  | | | |  | | |  | | |
|  |  | Low (mean-1 SD) | | 0.05 (0.02 – 0.08) | | | 0.001 | |  | | 0.01 (-0.04 – 0.06) | | | 0.664 | | |  | | | 0.05 (0.03 – 0.08) | | | <0.001 | | |  | | |  | | |  | | | |  | | |  | | |
|  |  | High v. low^d^ | | 0.09 (0.06 – 0.12) | | | <0.001 | |  | | 0.05 (-0.04 – 0.14) | | | 0.307 | | |  | | | -0.01 (-0.06 – 0.04) | | | 0.712 | | |  | | |  | | |  | | | |  | | |  | | |
|  | Physical bullying | | |  | | |  | |  | |  | | |  | | |  | | |  | | |  | | |  | | | 3.45 (2) | | | 0.178 | | | | 1.0 | | |  | | |
|  | Verbal bullying | | |  | | |  | |  | |  | | |  | | |  | | |  | | |  | | |  | | | 2.99 (2) | | | 0.224 | | | | 1.0 | | |  | | |
|  | Indirect bullying | | |  | | |  | |  | |  | | |  | | |  | | |  | | |  | | |  | | | 17.63 (2) | | | <0.001 | | | | 0.001 | | |  | | |
|  |  | High (mean+1 SD) | | 0.15 (0.13 – 0.17) | | | <0.001 | |  | | 0.07 (0.01 – 0.12) | | | 0.024 | | |  | | | 0.04 (0.01 – 0.07) | | | 0.022 | | |  | | |  | | |  | | | |  | | |  | | |
|  |  | Average (mean) | | 0.10 (0.08 – 0.12) | | | <0.001 | |  | | 0.03 (0.00 – 0.07) | | | 0.046 | | |  | | | 0.05 (0.03 – 0.07) | | | <0.001 | | |  | | |  | | |  | | | |  | | |  | | |
|  |  | Low (mean-1 SD) | | 0.06 (0.03 – 0.08) | | | <0.001 | |  | | 0.00 (-0.05 – 0.05) | | | 0.937 | | |  | | | 0.06 (0.03 – 0.09) | | | <0.001 | | |  | | |  | | |  | | | |  | | |  | | |
|  |  | High v. low^d^ | | 0.09 (0.06 – 0.12) | | | <0.001 | |  | | 0.06 (-0.02 – 0.15) | | | 0.144 | | |  | | | -0.02 (-0.07 – 0.02) | | | 0.349 | | |  | | |  | | |  | | | |  | | |  | | |
|  |  |  | |  | | |  | |  | |  | | |  | | |  | | |  | | |  | | |  | | |  | | |  | | | |  | | |  | | |

*Note:* SD, standard deviation; df, degrees of freedom; v., versus; CI, confidence interval; adj. β, standardized regression coefficients, continuous independent variables were standardized (mean=0, SD=1) for interpreting significant three-way interaction terms and examining the difference in associations between high (mean + 1 SD), average (mean), and low (mean – 1 SD) levels of exposure to bullying victimization within and across groups (service users, siblings, controls); *p*FWE , family-wise error-corrected p values were computed by multiplying the unadjusted p value by the total number of tests (*N*=8) to adjust signiﬁcance levels of likelihood ratio tests for three-way interactions.
^a^ Adjusted for age, sex, ethnicity, and level of education
^b^ Momentary stress was calculated by combining the ratings of six items assessing event-related, activity-related, and social stress
^c^ Three-way interaction term as included in the following model (with y_ij_ for negative affect or psychotic experiences as outcome variable): y_ij_  = β_0_ + β_1_(STRESS_ij_) + β_2_(BULLYING_j_) + β_3_(GROUP_j_) + β_4_(STRESS_ij_ × BULLYING_j_) + β_5_(STRESS_ij_ × GROUP_j_) + β_6_(BULLYING_j_ × GROUP_j_) + β_7_(STRESS_ij_ × BULLYING_j_ × GROUP_j_) + ε_ij_ (full model not shown - available upon request)

| **Table S4.** Sensitivity analysis: Association of stress with negative affect and psychotic experiences, by levels of bullying victimization in service users, siblings, and controls  with bipolar event-related stress item^a^ | | | | | | | | | | | |  |
| --- | --- | --- | --- | --- | --- | --- | --- | --- | --- | --- | --- | --- |
| ^d^ Difference in the magnitude of associations of momentary stress with negative affect and psychotic experiences between those exposed to high v. low levels of bullying victimization across groups (Δ high v. low): | | | | | | | | | | | |  |
|  | | *Service users vs. controls* | |  | *Siblings vs. controls* | |  | *Service users vs. siblings* | | | |  |
|  | | adj. β (95% CI) | p |  | adj. β (95% CI) | P |  | | adj. β (95% CI) | | p |  |
|  | |  |  |  |  |  |  |  | | |  |  |
| Δ high vs. low exposure levels  of bullying victimization across groups | |  | | | | | | | | | | |
|  | | Outcome: negative affect | | | | | | | | | | |
|  | |  |  |  |  |  |  | | |  |  |  |
| Momentary stress x bullying x group | |  |  |  |  |  |  | | |  |  |  |
|  | Overall bullying exposure | 0.19 (0.08 – 0.30) | 0.001 |  | 0.17 (-0.02 – 0.36) | 0.083 |  | | | 0.02 (-0.16 – 0.19) | 0.837 |  |
|  | Physical bullying | 0.40 (0.25 – 0.55) | <0.001 |  | 0.46 (0.26 – 0.65) | <0.001 |  | | | -0.05 (-0.20 – 0.09) | 0.459 |  |
|  |  |  |  |  |  |  |  | | |  |  |  |
| Outcome: psychotic experiences | | | | | | | | | | | |  |
|  | |  |  |  |  |  |  | | |  |  |  |
| Momentary stress x bullying x group | |  |  |  |  |  |  | | |  |  |  |
|  | Overall bullying exposure | 0.10 (0.04 – 0.16) | 0.002 |  | 0.06 (-0.05 – 0.16) | 0.285 |  | | | 0.04 (-0.05 – 0.13) | 0.409 |  |
|  | Indirect bullying | 0.12 (0.06 – 0.17) | <0.001 |  | 0.08 (-0.01 – 0.18) | 0.084 |  | | | 0.03 (-0.06 – 0.12) | 0.500 |  |
|  |  |  |  |  |  |  |  | | |  |  |  |
