# Supplementary material for: Bullying victimization and stress sensitivity in help-seeking youth: findings from an experience sampling study
Source: Eur Child Adolesc Psychiatry. 2020 May 13;30(4):591–605. doi: 10.1007/s00787-020-01540-5 (PMC8041697; doi:10.1007/s00787-020-01540-5)
Supplement: Supplementary file 5 — Supplementary file5 (DOCX 20 kb) [file 787_2020_1540_MOESM5_ESM.docx]

**Supplement 1: Results of exploratory analyses**

**Results**

***Association between momentary stressors and negative affect by bullying victimization and group***

We found no evidence that prior exposure to overall as well as specific types of bullying victimization modified the association of event-related with negative affect (Table S1). However, the association of activity-related stress and social stress with negative affect was amplified by physical bullying, but not by exposure to overall bullying victimization as well as other bullying types (i.e. verbal and indirect). Evidence for effect modification by levels of bullying exposure within and across groups was evidenced by statistically significant 3-way interaction effects (Table S1).

*Within-group comparisons*

Within groups, activity-related stress was associated with less intense negative affect in controls with high *vs.* low physical bullying levels (*adj. β*=-0.28, *p*<0.001), whereas no differences were observed in service-users (*adj. β*=0.05, *p*=0.120) and siblings (*adj. β*=0.04, *p*=0.619). In addition, social stress was associated with more intense negative affect in service users (*adj. β*=0.08, *p*=0.004) and less intense negative affect in controls (*adj. β*=-0.26, *p*=0.004), but no differences in siblings, when high *vs.* low physical bullying levels were compared.

*Between-group comparisons*

To investigate whether the impact of exposure to bullying victimization on stress sensitivity differed across groups, differences in magnitude of associations between those exposed to high *vs.* low levels of bullying victimization were examined across groups. The difference in magnitude of associations between activity-related stress and negative affect was greater in service users *vs.* controls (*adj. β*=0.33, *p*<0.001) and siblings *vs.* controls (*adj. β*=0.32, *p*=0.002), but not in service users *vs.* siblings (*adj. β*=0.01, *p*=0.891), when high vs. low levels of physical bullying were compared. In addition, there were significant differences in the magnitude of associations between social stress and negative affect by levels of physical bullying in service users vs. controls (*adj. β*=0.34, *p*<0.001) and siblings *vs.* controls (*adj. β*=0.39, *p*=0.001), but not service users *vs.* siblings (*adj. β*=-0.05, *p*=0.522).

***Association between momentary stressors and psychotic experiences by bullying victimization and group***

*Within-group comparisons*

We found no evidence that prior exposure to overall as well as specific types of bullying victimization modified the association of event-related and social stress with psychotic experiences (Table S2). However, we found evidence that activity-related stress was associated with more intense psychotic experiences in service users (*adj. β*=0.09, *p*<0.001), but not in siblings (*adj. β*=0.04, *p*=0.417) and controls (*adj. β*=-0.02, *p*=0.367) (Table S2) when high *vs.* low exposure levels to indirect bullying were compared, while no evidence for effect modification was found for overall bullying victimization as well as physical and verbal bullying.

*Between-group comparisons*

There were differences in the magnitude of associations of activity-related stress with psychotic experiences by high *vs.* low exposure levels to indirect bullying victimization comparing service users and controls (*adj. β*=0.11, *p*<0.001), but not service users and siblings (*adj. β*=0.05, *p*=0.280) and siblings and controls (*adj. β*=0.06, *p*=0.249).
